# S100A7 promotes the migration, invasion and metastasis of human cervical cancer cells through epithelial–mesenchymal transition

**DOI:** 10.18632/oncotarget.15329

**Published:** 2017-02-15

**Authors:** Tian Tian, Xukun Li, Zhen Hua, Jianlin Ma, Xiaowei Wu, Zhihua Liu, Hongyan Chen, Zhumei Cui

**Affiliations:** ^1^ Department of Obstetrics and Gynecology, The Affiliated Hospital of Qingdao University, Qingdao 266061, People's Republic of China; ^2^ State Key Lab of Molecular Oncology, National Cancer Center/Cancer Hospital, Chinese Academy of Medical Sciences and Peking Union Medical College, Beijing 100021, People's Republic of China; ^3^ Department of Anesthesiology, The Affiliated Hospital of Qingdao University, Qingdao 266061, People's Republic of China

**Keywords:** S100A7, cervical cancer, invasion, metastasis, EMT

## Abstract

S100A7 is an EF-hand calcium-binding protein that has been suggested to be implicated in cell proliferation, migration, invasion and tumor metastasis. However, its role in cervical cancer has not yet been fully clarified. The present study used immunohistochemistry analysis of S100A7 in clinical specimens of cervical cancer to show that S100A7 expression was significantly upregulated in cervical cancer tissues compared with normal cervical tissues and S100A7 expression in high grade cervical intraepithelial neoplasm (CIN) was significantly higher than cervical cancer. Statistical analysis showed that S100A7 expression was associated with tumor grade (*P* <0.01) and lymph node metastasis (*P* <0.05). Functional studies showed that overexpression of S100A7 in cervical cancer cells promoted migration, invasion and metastasis of cervical cancer cells without influencing cell proliferation. Furthermore, S100A7 was found to be secreted into the conditioned media and extracellular S100A7 enhanced cell migration and invasion. Mechanistically, S100A7 bound to RAGE and activated ERK signaling pathway. And S100A7 enhanced cell mesenchymal properties and induced epithelial–mesenchymal transition. In summary, these data reveal a crucial role for S100A7 in regulating cell migration, invasion, metastasis and EMT of cervical cancer and suggest that targeting S100A7 may offer a new targeted strategy for cervical cancer.

## INTRODUCTION

Cervical cancer is the fourth most common cancer in women, and the seventh overall, with an estimated 528,000 new cases in 2012 [1]. Human papillomavirus (most notably HPV16 and HPV18) have been definitely defined human carcinogens and their persistent infection in the cervix is established as a necessary cause for cervical cancer [2]. However, only a small fraction of infections that persist may progress to cervical cancer, indicating that many other factors contribute to the progression of cervical cancer. Epithelial–mesenchymal transition (EMT), is a potential mechanism by which cancer cells to depart from the primary tumor, invade surrounding tissue and disseminate to distant organs, resulting in invasion and metastasis. Diverse factors secreted by the cancer cells and host cells in their local microenvironments may trigger the molecular events that are associated with the EMT program [3]. A number of findings indicate that multiple molecules were involved in the regulation of EMT in cervical cancer. For instance, AEG-1, Sam-68, FTS, miR-361-5p induce EMT, while LMX-1, SFPR1 and miR-155 suppress EMT in cervical cancer [4]. Therefore, further identifying and characterizing key factors involved in EMT in cervical cancer is important for developing new therapeutic approaches.

The S100 gene family is the largest subfamily of calcium binding proteins of EF-hand type. S100 proteins have multiple intracellular functions including regulation of cell cycle progression, cell proliferation, migration, invasion, phosphorylation, cytoskeletal components and regulation of transcriptional factors etc. S100 proteins can also be secreted from cells to exert extracellular functions. Some of the extracellular functions may be mediated by binding to the receptor for advanced glycation end products (RAGE) and triggerring RAGE-mediated cellular signaling which involves in MAP Kinase, NF-κB, and phosphatidylinositol 3-kinase (PI-3K)/AKT signaling pathway [5]. S100A7, also called Psoriasin, is a member of the S100 protein family that was originally identified as one of the most abundant proteins in psoriatic keratinocytes [6]. It is found aberrantly expressed in multiple types of cancer including oral squamous cell carcinomas, breast cancer, prostate cancer, osteosarcoma, head and neck cancer, lung cancer and ovarian cancer [7–13]. Functional investigation demonstrated that S100A7 regulated cell proliferation, migration, invasion, angiogenesis and metastasis [7, 14, 15]. S100A7 can be induced by inflammatory cytokines, including oncostatin M (OSM), interleukin (IL)-6 [16]. In turn, S100A7 induced expression of inflammatory cytokines/chemokines [15]. S100A7 modulates a series of genes linked to the immune response, suggesting that S100A7 may act as host conditions or stromal factors [17]. And soluble S100A7 was shown to induce chemotaxis in leukocytes by binding to RAGE. In addition, S100A7 may enhance breast cancer growth and metastasis through upregulating proinflammatory pathways and recruiting tumor-associated macrophages (TAMs) [15]. Mechanistically, S100A7 enhances survival of breast cancer cells by binding to c-Jun activation domain-binding protein 1 (Jab1) and increasing activity of NF-κB and p-Akt [18]. In contrast, S100A7 exhibits tumor suppressor capabilities through down-modulation of the β-catenin pathway [7, 8]. Knockdown of S100A7 suppresses lung cancer growth in part by attenuating NF-κB activity, and S100A7 promotes the migration and invasion of osteosarcoma cells and enhances the activity of MMP2 and MMP9 through RAGE [10, 19].

However, the role of S100A7 in cervical cancer and its underlying molecular mechanisms are still not clarified. In the present study, we firstly detected the expression of S100A7 in cervical cancer tissues and analyzed the correlation between S100A7 expression and clinicopathologic characteristics. Furthermore, we investigated the biological functions and the underlying molecular mechanism by overexpression of S100A7 in cervical cancer cells. Our data indicated immunoactivity of S100A7 on cervical cancer tissues correlates with tumor grade and lymph node metastasis, and S100A7 was involved in cervical carcinogenesis by promoting migration, invasion, metastasis and epithelial-mesenchymal transition. Thus, our study implicated that S100A7 played an important role in the pathogenesis of cervical cancer and S100A7 might be a novel valuable therapeutic target for cervical cancer.

## RESULTS

### S100A7 expression correlates with histological type of cervical cancer, tumor grade and lymph node metastasis

**Figure 1:**
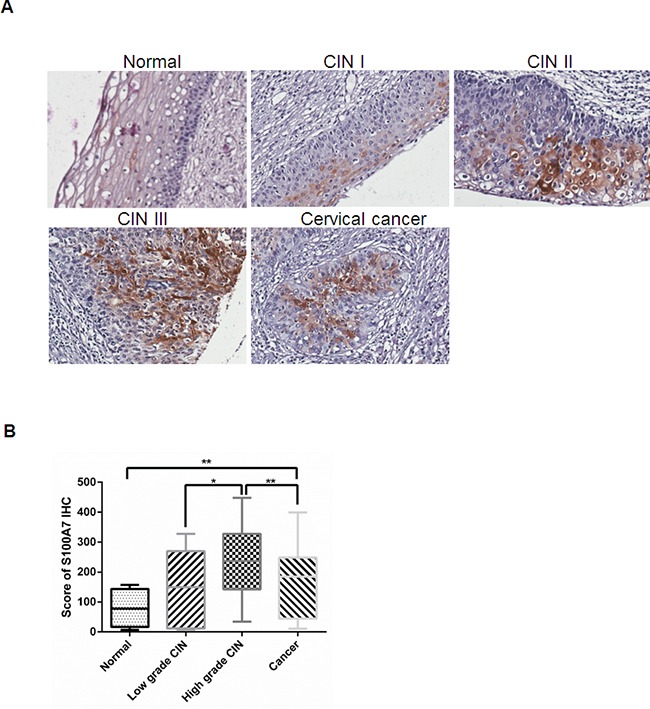
S100A7 expression in human normal cervical tissue, CIN and cervical cancer specimens **A**. Representative immunohistochemistry staining images of S100A7 in cervical specimens. S100A7 is expressed incrementally in normal cervical tissue, cervical cancer and high grade CIN. **B**. Score of S100A7 immunohistochemistry staining in normal cervical tissue, CIN and cervical cancer.

Previous studies revealed that S100A7 was differentially expressed in multiple cancers. However, the correlation between S100A7 expression and clinicopathological features in cervical cancer has not been studied. Thus we performed Immunohistochemistry (IHC) of formalin-fixed, paraffin-embedded tissue microarrays composed of 24 samples of normal cervical tissue, 88 cases of CIN and 51 primary cervical carcinomas (Table 1). The images were collected at 20× magnification using Aperio image scope and the expression of S100A7 was analyzed using Aperio ImageScope software. S100A7 was observed mainly in cell membrane and cytoplasm, nuclei staining was also observed in strong positive cells (Figure 1A). And S100A7 expression displayed a scattered or a patchy distribution in all positively stained tissues. S100A7 expression was higher in cervical cancer tissues than that in normal tissues (*P* < 0.01). Moreover, S100A7 expression was increased in high grade CIN compared with cervical cancer (*P* < 0.01) (Figure 1B).

**Table 1:** The correlation between S100A7 expression and clinicopathologic characteristics in IHC analysis

Clinical Variable	Relative expression^a^ of S100A7 and *p*-value of different categories^b^		
	N	Median	*p*-value
**Diagnostic category**			<0.001
Normal	24	77.76	
Low grade CIN	10	149.67	
High grade CIN	78	237.06	
Cancer	51	185.01	
**Histological type**			0.017
Squamous carcinoma	47	200.53	
Adenocarcinoma	4	22.47	
**FIGO stage**			0.795
I	38	176.25	
II+ III	13	191.83	
**Tumor grade**			0.007
G1	4	299.28	
G2	34	205.34	
G3	13	91.16	
**Tumor size(cm)**			0.901
<4cm	36	164.82	
≥4cm	15	210.16	
**Lymph node metastasis**			0.033
Negative	36	155.43	
Positive	15	247.81	

^a^Median of relative expression

^b^Mann-Whitney *U* and Kruskal-Wallis nonparametric test were used for comparing different groups

To further determine whether S100A7 overexpression is linked to clinicopathological features, 51 cervical cancer specimens were grouped according to their histological type, FIGO stage, tumor grade, histological grade, tumor size, lymph node metastasis. The statistic results showed that S100A7 immunoreactivity significantly correlated with histologic subtype (*P* =0.017), tumor grade (*P* = 0.007), and lymph node metastasis (*P* = 0.033) (Table 1).

### S100A7 overexpression increases cell migration and invasion in cervical cancer cells

**Figure 2:**
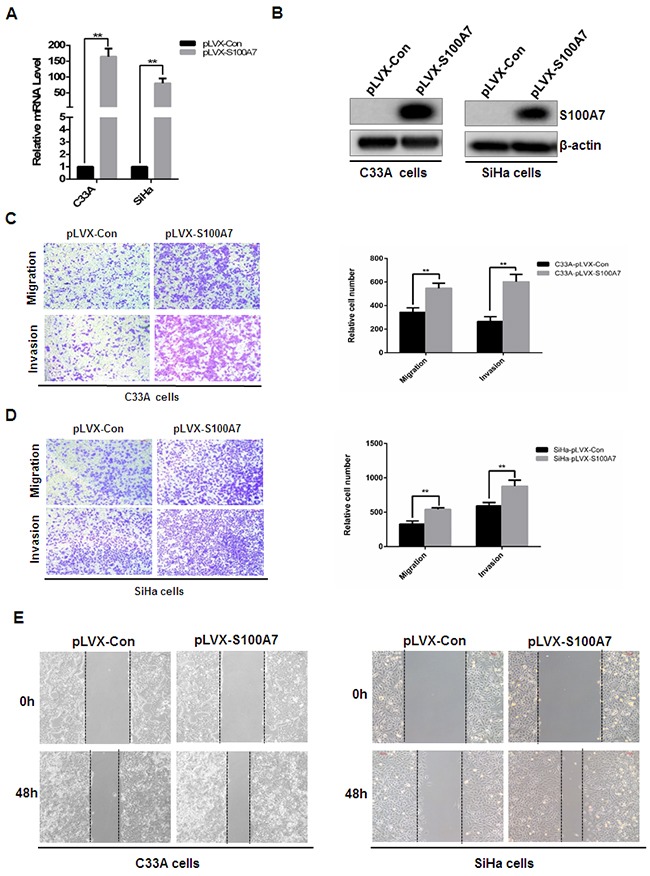
S100A7 promotes cervical cancer cell migration and invasion **A&B**. Establishment of stable cell lines of ectopic expression of S100A7. C33A and SiHa cells were infected with pLVX-Con and pLVX-S100A7 lentivirus, stable cells were established by Geneticin (G418) selection for about 2 weeks. Cells were harvested, S100A7 expression was detected by qRT-PCR (A: [mean (n=2) ± SD; 2-sided t test; ** *P*<0.01, normalized to β-actin]) and Western Blot. **C&D**. Transwell migration and Matrigel invasion assays were performed in S100A7-overexpressed cells and their corresponding control cells. The stained cells were manually counted from 4 randomly selected fields and normalized with cell proliferation [2-sided *t* test; **P < 0.01]. Representative image and quantitative results of cell migration and invasion were shown (C. C33A cells; D. SiHa cells; ×10, bars:100μm). **E**. The effect of S100A7 on cell migration was examined in wound-healing assay. Confluent cells were scratched and photographed at time 0h and 48h (**Left panel**. C33A cells; **Right panel**. SiHa cells. ×10, bars:100μm).

On the basis of the IHC analysis of S100A7 expression in cervical cancer, we speculate that S100A7 plays an important role in tumorigenesis and cancer progression. We set out to investigate the potential role of S100A7 in the development of a malignant phenotype in cervical cancer cells by modulating intracellular S100A7 expression. We firstly examined mRNA and protein expression levels of S100A7 in the four common cervical cancer cells including C33A, HeLa, SiHa and CaSki and found that S100A7 was expressed at a low level in the four cell lines. We therefore established stable S1007-overexpressed cells using lentiviral-mediated gene delivery in C33A and SiHa cells. S100A7 expression was assessed using real-time quantitative reverse transcription PCR (qRT-PCR) and Western Blot analysis and an average of 100 fold increase in S100A7 was detected in cells transfected with S100A7 compared with cells transfected with vector alone (Figure 2A&2B). Previous studies demonstrated that S100A7 acts as a dual regulator of cell proliferation. [7, 20]. To detect the effect on cervical cancer cell proliferation of S100A7 overexpression, cell proliferation was assessed by CCK-8 assay. The rate of proliferation of S100A7-overexpressed cells was not significantly different from that of control cells (Supplementary Figure 1A&1B). Cell cycle distribution was further detected by FACS. Consistent with cell proliferation data, S100A7 has no significant effect on cell cycle distribution (Supplementary Figure 1C&1D). Our IHC results indicated that S100A7 expression is significantly correlated with lymph node metastasis. These phenomena led us to hypothesize that S100A7 may also be involved in the migration/invasion of cervical cancer cells. To test this hypothesis, cell migration and invasion assays were performed. As expected, S100A7 overexpression significantly promoted migration and invasion of C33A (Figure 2C), and SiHa cells (Figure 2D). Similarly, the capability of wound-healing is obviously increased in S100A7-expressing C33A (Figure 2E) and SiHa (Figure 2F) cells compared with control cells. These results indicated that S100A7 may play an inducer of cell migration/invasion in cervical cancer.

### S100A7 was secreted into the conditioned media and extracellular S100A7 enhanced cell migration and invasion of cervical cancer cells

**Figure 3:**
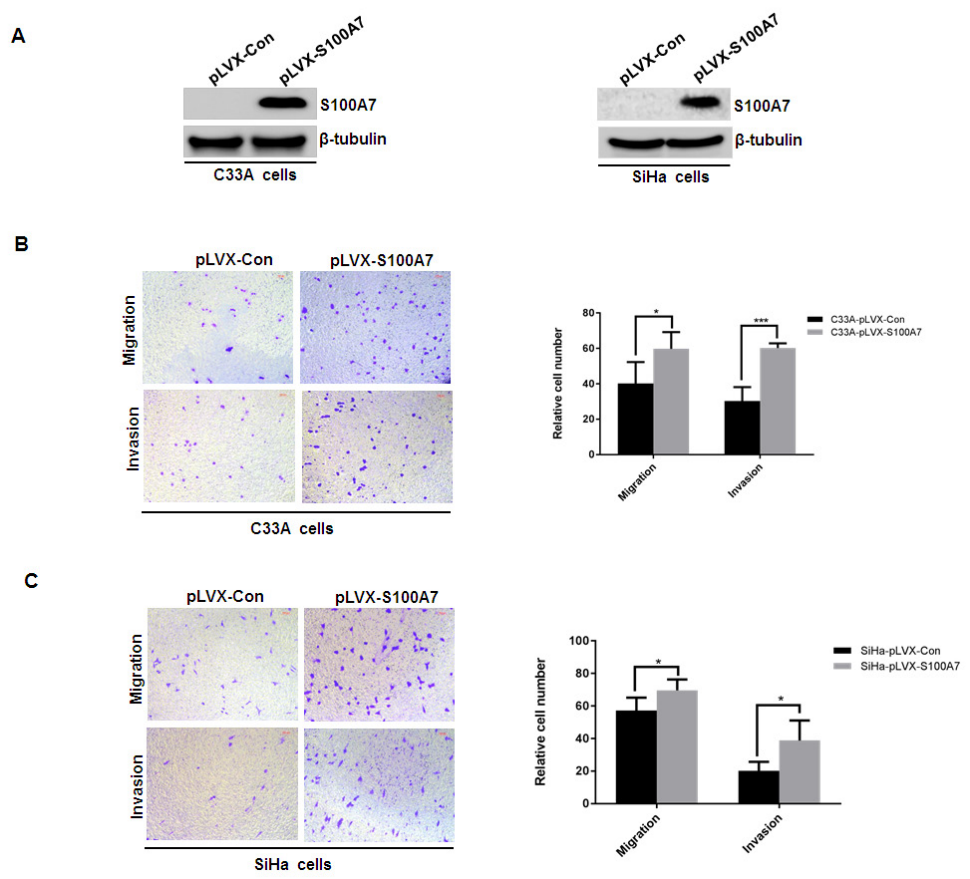
S100A7 is secreted and acts as a chemotactic factor of cell migration and invasion **A**. Cells were cultured in serum-free medium for 2 days, then the medium was collected, fractioned, followed by Western Blot analysis. **B&C**. Transwell migration and Matrigel invasion assays were performed in C33A and SiHa cells. Cell suspension was placed into the upper chamber in 0.1 ml of DMEM serum-free medium, and conditioned medium from S100A7-overexpressed C33A and SiHa cells and their corresponding control cells was placed in the lower chamber as a chemoattractant. The stained cells were manually counted from 4 randomly selected fields and normalized with cell proliferation [2-sided *t* test; *P < 0.05; ***P < 0.001]. Representative image and quantitative results of cell migration and invasion were shown (B. C33A cells; C. SiHa cells; ×10, bars:100μm).

Since S100A7 was found to be a secreted chemotactic factor [16]. We next examined whether S100A7 can be secreted into the conditioned media in S100A7-overexpressed C33A and SiHa cells. Cells were cultured in serum-free medium for 2 days, then the medium was collected, fractioned, followed by Western Blot analysis. And S100A7 expression was observed in the supernatant of S100A7-overexpressed C33A and SiHa cells. In contrast, no expression of S100A7 was detected in conditioned media from control cells (Figure 3A). Furthermore, we analyzed the biological activity of secreted S100A7. Chemotactic migration and invasion were performed using conditioned media from S100A7-overexpressing C33A and SiHa cells and their corresponding control cells. The results showed that conditioned media from S100A7-overexpressed cells significantly enhanced migration and invasion of C33A and SiHa cells (Figure 3B&3C). These data suggested that extracellular S100A7 acts as an inducer of cell migration and invasion.

### S100A7 interacts with RAGE, activates ERK signaling and enhances cell migration and invasion via the interaction with RAGE

**Figure 4:**
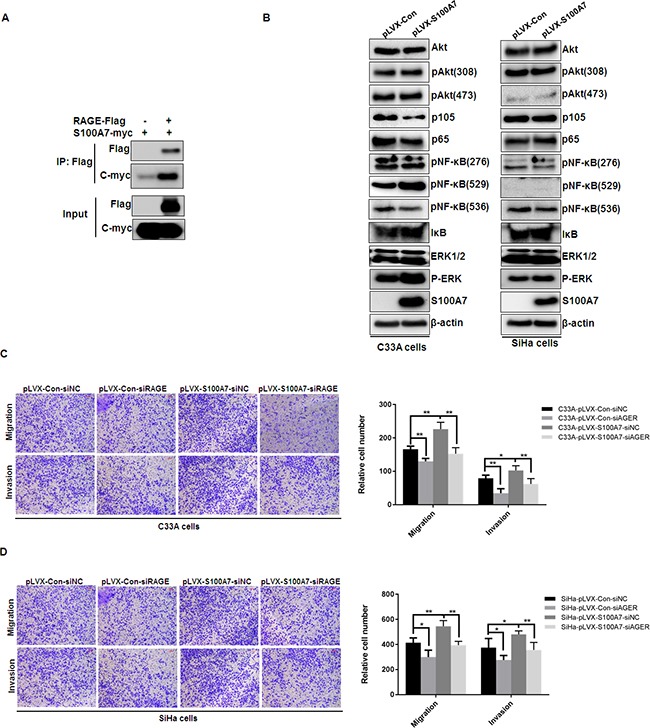
S100A7 interacts with RAGE, stimulates ERK pathway and RAGE mediates S100A7-induced migration and invasion in C33A and SiHa cells **A**. HEK293 cells were transiently co-expressed with pcDNA3-Flag and pcDNA3-Flag-RAGE with pcDNA3-myc-S100A7. Cell extracts were immunoprecipitated separately with specific antibody against Flag, and the associated Flag-RAGE and myc-S100A7 were examined by Western blotting using tagged antibodies, respectively. **B**. S100A7-expressing cells were found to have higher levels of phospho-ERK (without any change in total ERK levels). In comparison, the levels of phospho-Akt and phospho-NF-κB had no obvious change. **C**. qRT-PCR verification of RAGE knockdown by two independent siRNAs in C33A and SiHa cells. **D&E**. RAGE knockdown suppressed migration and invasion in S100A7-overexpressed C33A and SiHa cells and their corresponding control cells. The stained cells were manually counted from 4 randomly selected fields and normalized with cell proliferation [2-sided *t* test; * *P* < 0.05; **P < 0.01]. Representative image and quantitative results of cell migration and invasion were shown (D. C33A cells; E. SiHa cells; ×10, bars: 100μm).

Prior studies have identified that RAGE as the S100A7 receptor and S100A7 induced cell growth, migration, invasion and metastasis through the interaction with RAGE [10, 14]. To further validate the interaction between S100A7 and RAGE, pcDNA3-Flag and pcDNA3-Flag-RAGE with pcDNA3-myc-S100A7 were co-expressed into HEK293 cells and co-immunoprecipitation experiment was performed. After immunoprecipitating RAGE, we detected the presence of S100A7 (Figure 4A). Our results suggested that S100A7 can bind to RAGE *in vivo*. As a general receptor of S100 proteins, RAGE-mediated cellular signaling was involved in phosphatidylinositol 3-kinase (PI-3K)/AKT, NF-κB, and MAP Kinase signaling pathway [5]. We therefore assessed Akt, NF-κB, and ERK expression and phosphorylation status in S100A7-overexpressed C33A and SiHa cells and their corresponding nctrol cells. The results demonstrated that S100A7 overexpression stimulated ERK signaling, but has no obvious effect on NF-κB and Akt signaling pathway (Figure 4B). These results promoted us to further investigate whether RAGE mediates S100A7-induced cell migration and invasion. We suppressed RAGE expression by siRNA transfection and assessed the effect of RAGE knockdown on S100A7-induced cell migration and invasion. RAGE expression was efficiently inhibited by two independent siRNAs (Figure 4C). And we selected one RAGE siRNA to perform transwell assay. The results showed that suppression of RAGE efficiently inhibited cell migration and invasion in S100A7-overexpressed C33A and SiHa cells and their corresponding control cells (Figrue 4D&4E). These data suggested that S100A7 enhanced cell migration and invasion is at least partially mediated by RAGE.

### S100A7 mediates epithelial-to-mesenchymal transition (EMT)

**Figure 5:**
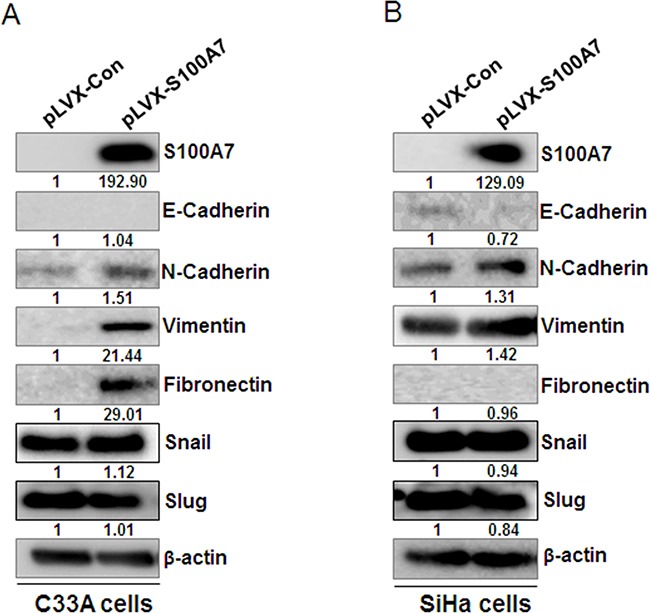
S100A7 induces EMT in cervical cancer cells Western Blot showed the protein level of epithelial marker E-cadherin and mesenchymal markers (N-Cadherin, Vimentin, Fibronectin) and EMT transcription factors Snail and Slug after overexpression of S100A7 in C33A cells **A**. and SiHa cells **B**. β-actin is used as a loading control.

In a process of EMT, cancer cells employ developmental processes to gain migratory and invasive properties [21]. The regulation of EMT is also reported to participate in the progression of cervical squamous cell carcinoma [22]. It has been reported that RAGE increased MEK-EMT signaling and promoted migration, invasion and metastasis. And RAGE overexpression increased the expression of EMT markers including matrix metalloproteinases, MMP-2, MMP-9 and vimentin, while concurrently reducing the expression of epithelial E-cadherin and ZO-1. Furthermore, RAGE overexpression increased the expression of EMT transcription factors Slug and Twist1 [23]. Since S100A7 is an important ligand of RAGE, we speculate that S100A7-RAGE-binding is involved in the regulation of EMT. We therefore detected the expression of epithelial markers (such as E-cadherin) and mesenchymal markers (such as N-cadherin, Vimentin, and Fibronectin) after overexpression of S100A7 by Western Blotting. Meanwhile, we also assessed the expression of EMT transcription factors including Snail and Slug. The results indicated that E-cadherin was downregulated while N-cadherin, Vimentin, and Fibronectin were upregulated except for the null detection of E-cadherin in C33A cells and Fibronectin in SiHa cells, whereas Snail and Slug levels were unchanged (Figure 5A&5B). These results illustrated that S100A7 may act as an inducer of EMT, thereby enhancing cell migration and invasion.

### S100A7 enhances metastasis of cervical cancer cells *in vivo*

**Figure 6:**
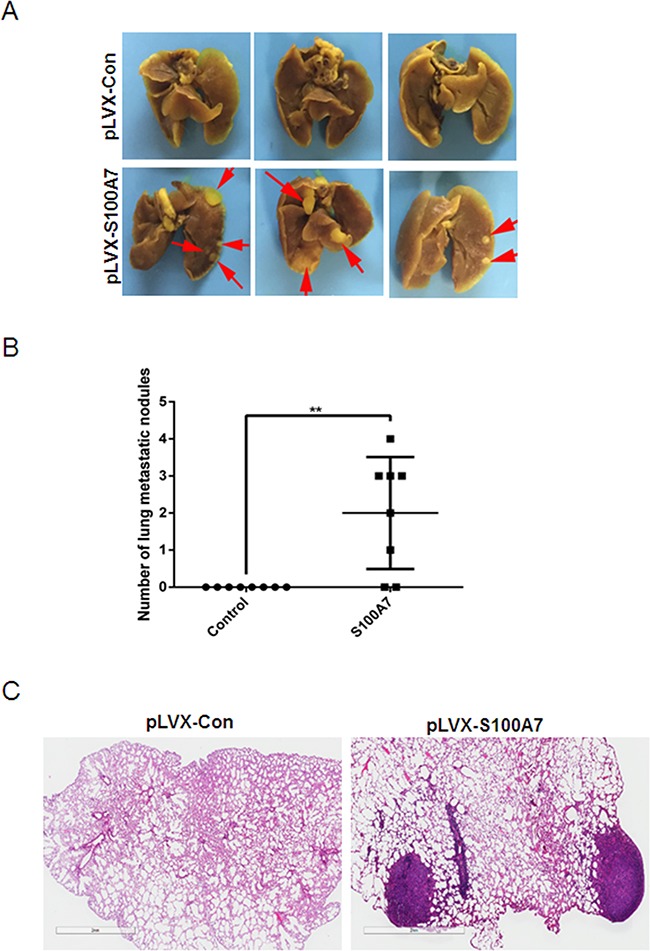
Macroscopic and microscopic features of the lung metastasis by SiHa cells SiHa cells were injected into the tail vein of nude mice, and experimental lung metastasis was determined on week 15. **A**. The metastatic tumors was visible (marked by red arrows) in the mice-injecting S100A7-overexpressed cells. In contrast, no metastatic tumors were found in the control group. **B**. Number of lung metastatic nodules. **C**. H&E staining of the paraffin-embedded sections of lung metastatic nodules (×1, bars: 2mm).

To further determine whether S100A7 promotes *in vivo* metastasis, we performed intravenous injection of S100A7-overexpressed SiHa cells and control cells. In line with *in vitro* results, S100A7 overexpression significantly increased the accumulation of SiHa cells in the lungs of inoculated athymic mice (Figure 6A&6B). Histological staining was performed to further confirm the presence of lung metastases (Figure 6C). These data confirmed the essential role of S100A7 for metastatic growth of cervical cancer cells.

## DISCUSSION

Cervical cancer is a common cancer in women worldwide especially in developing country. High-risk human papillomavirus is the predominant factor in the development of the precursors of cervical cancer. However, only a small number of HPV positive patients develop cancer, indicating many other factors contributing to the progression of cervical cancer. S100A7, also known as Psoriasin, belongs to the S100 multigenic family of calcium-modulated proteins of the EF-hand type and was originally identified in psoriatic keratinocytes [6]. S100A7 has been suggested to be implicated in a variety of biologic events closely related to tumorigenesis and cancer progression. However, information is limited about the possible biologic significance of the altered expression of S100A7 during cervical cancer development. In this study, we performed IHC analysis of S100A7 in normal cervical tissue, cervical cancer and CIN and found that the staining of S100A7 was scattered, suggesting that S100A7 displays the cellular heterogeneity in cervical cancers, which is supported by previous study that S100A7 exhibits heterogeneous and inducible characteristic in squamous cell carcinomas (SCC) [24]. Moreover, S100A7 expression is higher in cervical cancer compared with normal cervical tissue. Notably, it is highly expressed in high-grade squamous intraepithelial lesion, indicating its important role in the early stages of cervical tumor progression. Similar to the role of S100A7 in oral squamous cell carcinomas (OSCC), with high expression in pre-invasive, well-differentiated and early-stage oral squamous cell carcinomas, but not in non-invasive, poorly differentiated, late-stage tumors [7]. Consistently, other studies also showed that S100A7 expression was relatively low in normal, benign and atypical hyperplastic proliferative ductal lesions, high in the pre-invasive ductal carcinoma in situ (DCIS), but reduced in invasive carcinomas [25–28]. In addition, S100A7 is also expressed in the normal cervical tissue, and many cervical koilocyte were found in our normal cervical tissue samples which may be caused by HPV infection. It is reported that S100A7 had a significantly higher expression in condylomata acuminate which is related to HPV infection [29]. So we speculated that S100A7 expression in normal cervical tissue may have a relationship with HPV infection. Unfortunately, we did not test for HPV in the samples and could not assess the interactions of S100A7 with HPV in this study. As a pro-inflammatory protein, S100A7 has been suggested to be induced by the proinflammatory cytokines [16]. S100A7 overexpression in cervical cancer may also be due to inflammatory cytokines stimulation, which needs further investigation in the future.

Furthermore, we revealed a significant correlation between S100A7 expression and histologic subtype, tumor grade, and lymph node metastasis. S100A7 expression can serve as a biomarker for identifying dysplastic lesions at high risk of cancer development [20, 30]. And S100A7 overexpression, as an important risk factor, associated with reduced disease-free survival of OSCC patients [11]. We also examined the relationship between S100A7 expression and patient outcome. Kaplan-Meier plots demonstrated that there was no significant difference between S100A7 expression and survival of patients (data not shown). This could be attributable to the relatively small sample size. In future studies, we will increase sample size to evaluate the correlation between S100A7 expression and the survival of the patients.

Several lines of evidence demonstrated that S100A7 is involved in the regulation of cell proliferation, migration, invasion, angiogenesis and metastasis of numerous malignant tumors. However, its biologic role particularly in cervical cancer remains to be defined. In this study, we examined the functions of S100A7 by lentiviral-mediated overexpression in cervical cancer cells. The results demonstrated that S100A7 overexpression dramatically promoted cell migration, invasion and metastasis of cervical cancer cells. However, S100A7 does not influence cell proliferation and cell cycle progression, the most two important features among the malignant cell behavior. One possible explanation is that the impact of S100A7 on cell proliferation may vary in different cell types.

S100A7 was found to be secreted, extracellular S100A7 promotes migration and invasion osteosarcoma cells via RAGE. And the level of S100A7 protein in serum may serve as a potential marker in lung cancer and ovarian cancer [12, 13]. Correlating with its secreted nature, there is evidence that S100A7 can act as a chemotactic factor. In this study, we showed that S100A7 was secreted in the supernatant of S100A7-overexpressing cells and conditioned media of S100A7-overexpressing cells enhanced migration and invasion of cervical cancer cells. Our studies demonstrated that S100A7 promoted cervical cancer cell migration and invasion as a secreted chemotactic factor.

Mechanistic investigations have defined RAGE as the S100A7 receptor and RAGE mediated S100A7 induced cell growth, migration, invasion and metastasis by modulating the tumor microenvironment [10, 14]. In this study, we further confirmed the interaction between S100A7 and RAGE by Co-immunoprecipitation assay and demonstrated S100A7 can interact with RAGE *in vivo*. Previous studies have revealed that S100A7 bound to RAGE to activate ERK, NF-κB and S100A7 interacted with Jab1 to increase activity of NF-κB and p-Akt [14, 18]. We therefore assessed ERK, Akt and NF-κB expression and phosphorylation status in C33A and SiHa cells overexpressing S100A7 and their corresponding control cells. The results demonstrated that S100A7 overexpression stimulated ERK signaling, but has no obvious effect on NF-κB and Akt signaling pathway. Furthermore, knockdown of RAGE by siRNA transfection significantly suppressed S100A7-induced cell migration and invasion, indicating that S100A7 promotes cervical cancer cell migration and invasion at least partially through RAGE. These data suggest that S100A7 is associated with induction of phospho-ERK and that the induction of migration/invasion pathways may be dependent on S100A7-RAGE interaction.

EMT is a biological process that allows a polar-ized epithelial cell to undergo a series of biochemical changes that enable it to acquire a mesenchymal cell phenotype, thereby enhancing tumor migration, invasion and metastasis [31]. As important ligands of RAGE, several S100 proteins have been reported to act as regulators of EMT and be involved in cell migration, invasion and tumor metastasis. S100A8/A9 enhanced cell mesenchymal properties and induced epithelial-mesenchymal transition dependent on binding to RAGE. Mechanistically, S100A8/A9 stabilized Snail through the NF-κB signaling pathway [32]. S100A4 is commonly used as a mesenchymal cell marker and promotes tumor metastasis dependent on the interaction with RAGE [33]. S100A6 induces EMT and promotes cell migration and invasion via β-catenin activation [34]. Our previous study has shown that S100A14 can act as a mediator of EMT and promote cervical cancer cell migration and invasion [35]. Present data extend prior work and indicate that S100A7 drives upregulation of EMT markers N-Cadherin, Vimentin, and Fibronectin and loss of epithelial markers E-cadherin, thereby inducting EMT, promoting cervical cancer cell migration and invasion by inducing EMT. Future research delineates the details of how S100A7 regulates its downstream targets, and how these interactions influence the migrative/invasive properties remains to be determined.

In summary, our present research showed that S100A7 induces EMT and promotes cell migration, invasion and metastasis of cervical cancer cells. And S100A7 overexpression might serve as a useful marker for estimating the risk of cervical dysplastic lesions progressing to malignancy.

## MATERIALS AND METHODS

### Tissue samples

Tissue specimens from 51 cervical cancers, 88 cervical intraepithelial neoplasms (CIN), and 24 normal cervical tissues were included in this study and were obtained from the Affiliated Hospital of Qingdao University during the year of 2004 -2008. The clinical and clinicopathological classifications and staging were determined according to the 2009 FIGO criteria. None of patients received radiotherapy or chemotherapy before cervical biopsy and surgical resection. This study was approved by ethics committee of the Affiliated Hospital of Qingdao University. Clinical information pertaining to the samples is summarized in Table 1. Histological diagnosis was confirmed by two pathologists respectively.

### Immunohistochemistry staining

The PV-9000 kit was purchased from Beijing Zhongshan Golden Bridge Biotechnology Company. Mouse monoclonal antibody against human S100A7 (Novus Biologicals, Littleton, CO, USA) was diluted 1: 75. All procedures were implemented according to the manufacturer's instructions. For negative controls, sections were treated with 0.01 mol/L phosphate-buffered saline instead of primary antibodies. Evaluation and quantification of immunohistochemistry was performed as previously described [36]. All slides were digitalized and were evaluated on an ImageScope software (Aperio Technologies) using the positive pixel counting algorithm. This algorithm measures the percentage of positivity by area and the average intensity of positive staining.

### Cell cultures

Cervical cancer-derived cell lines (C33A, HeLa, SiHa and Caski) and HEK293T cells were purchased from the American Type Culture Collection (ATCC; Manassas, VA). C33A, HeLa, SiHa and HEK293T cells were maintained in Dulbecco's modified Eagle's medium and Caski cells were grown in RPMI1640 medium supplemented with 10% fetal bovine serum (FBS; Invitrogen, Carlsbad, CA), penicillin (100 U/mL) and streptomycin (100 U/mL). Cell cultures were maintained at 37°C in a humidified atmosphere containing 5% CO_2_.

### Lentiviral construction, production and transduction

Full-length cDNA of human S100A7 was cloned between *Xho* I and *Bam*H I of pLVX-IRES-Neo to generate the constitutive lentiviral vector. 6×10^6^ 293 T cells were seeded in 100 cm^2^ plates. 24 hours later, 7.5 μg pLVX-IRES-Neo or pLVX-IRES-Neo-S100A7 lentiviral vector, 6.5 μg pCMV Δ8.91, 3.5μg VSVG and 2.5μg PLP2 were transfected into 293T cells using Lipofectamine 2000 (Invitrogen, CA, USA). 8 hours later, the medium was removed and replaced with fresh culture medium. 48 hours post transfection, the virus-containing medium was harvested and subsequently pre-cleaned with a 3,000 g centrifuge and a 0.45μm filtration (Millipore). For lentiviral transduction, 2∼3×10^5^ cells/well were seeded in 6-well culture plates and infected the following day with lentiviruses. To generate stable cell lines, cells were selected for two weeks with 450μg/ml Geneticin.

### SiRNA transfection

Cells were transfected with siRNAs (40 nmol/L) by HiperFect (Qiagen) following the manufacturers’ protocol. RAGE siRNAs were as follows. 5′-GCAGTAGTAGGT GCTCAAA-3′ and 5′-TGGGAAGCCAGAAATTGTA-3′.

### Quantitative real-time RT-PCR

Total RNA was generated using TRIzol reagent (Invitrogen) and cDNA was synthesized using a High Capacity cDNA Reverse Transcription Kit (Applied Biosystems, Foster City, CA). The SYBR Green PCR Master Mix and the StepOne Plus^TM^ Real-Time PCR System from Applied Biosystems was used in the quantitative real-time PCR following the manufacturer's instructions. The primers are as follows: S100A7-F: CATCCTTCTACTCGTGACGCT; S100A7-R: CGATCATGCCTATTATGGACC; β-actin-F: GGCGGCACCACCATGTACCCT; β-actin-R: AGGGG CCGGACTCGTCATACT.

### Western blotting

Cells were lysed with cell lysis buffer, then protein extracts were separated with 10% SDS-PAGE, transferred to polyvinylidene difluoride membrane. The membrane was blocked with 5% nonfat milk for 1 hour at room temperature. Membranes were then incubated with primary antibody overnight at 4°C and secondary antibody diluted 1:5000 for an hour. The membranes were washed extensively in TBS, and were detected by enhanced chemiluminescence detection system (Pierce, Rockford, IL, USA). Anti-S100A7 (47C1068, Novus), Akt1/2/3(sc-65487, Santa Cruz Biotechnology), p-AKT 473 (sc-7985-R, Santa Cruz Biotechnology), p-AKT 308 (sc-16646-R, Santa Cruz Biotechnology), NFκB-p65 (phospho Ser276)(RLP0187, Ruiying Biological), NFκB-p65 (phospho Ser529)(RLP0190, Ruiying Biological), NFκB-p65 (phospho Ser536) (RLP0191, Ruiying Biological), NFkB p65 (RLM3053, Ruiying Biological), NFκB-p105/p50(RLT3101, Ruiying Biological), ERK1/2 (RLT1625, Ruiying Biological), p-ERK(sc-7383, Santa Cruz Biotechnology), E-Cadherin (ab40772, abcam), N-Cadherin (610921, BD Biosciences), Vimentin (sc-6260, Santa Cruz Biotechnology), Fibronectin (1573-1, Epitomics), Snail (3879S, Cell Signaling Technology), Slug (9585T, Cell Signaling Technology) antibodies were used. β-actin antibody (A5316, Sigma) was used to test for equal loading.

### Conditioned medium

The normal medium was removed from 80% confluent cells and cells were washed three times with PBS before addition of serum-free medium. Tumor cells were cultured in serum-free medium for 2 days. Then the medium was collected and centrifuged at 3000×g for 10 min to remove cell pellet. The medium was fractioned at 3 KDa MWCO (Millipore) at 4000×g for 50 min and filtered by passing through a 0.22μm filter (Millipore) before use or stored at -80°C.

### Cell proliferation assay

The stable transfected cells were seeded into 96-well plates (100 μl/well; 3000 cells/well) and incubated at 37°C with 5% CO_2_. 10 μl of CCK-8 solution was added into each well at four time point followed by incubation for 1h. The change in absorbance at 450 nm was spectrophotometrically measured. The experiment was done 3 times.

### Fluorescence-activated cell sorting analysis

Cells from the flask were detached by Trypsin-EDTA. After washing by ice-cold PBS, the cells were fixed in methanol at 4°C overnight. The fixed cells were resuspended in 500μl of PI staining solution (50 μg/mL propidium iodide, 100 μg/mL RNase, and 0.1% Nonidet P-40) and incubated for 30 minutes at 37°C. The result was analyzed on a FACS Calibur cell flow cytometer (Becton Dickinson).

### Cell migration and invasion assay

Migration and invasion assays were carried out using a Boyden chamber with 8μm pore polyethylene terephthalate (Costar, Cambridge, MA, USA) according to manufacturer protocol. The chambers were coated with Matrigel (BD Biosciences, San Jose, USA) when cell invasion assay was done. Briefly, cells (5×10^4^to 1×10^5^) were plated in the upper chamber in serum free media. The bottom chamber contained DMEM media with 10% FBS. Following 24∼48 hours, the bottom of the chamber insert was fixed and stained with 0.3% crystal violet. Cells on the stained membrane were counted under a dissecting microscope. Each membrane was divided into four quadrants and an average from all four quadrants was calculated. Each migration or invasion assay was done in biological triplicates. When exploring the biological activity of secreted S100A7, conditioned medium was placed in the lower chamber as a chemoattractant, meanwhile, the cell suspension was placed into the upper chamber in 0.1 ml of serum-free DMEM medium (1×10^5^ cells per filter) and allowed to migrate and invade through the insert membrane for 48 h.

### *In vitro* wound-healing assay

Cells were plated and grown overnight to reach confluence in 6-well plate, monolayers of cells were scratched using a pipette tip. Cells were washed to remove cellular debris and allowed to migrate for 48 hours. Images were taken at 0 h and 48 h after wounding using an inverted microscope.

### Co-immunoprecipitation

2μg Plasmid pcDNA3-Flag and pcDNA3-Flag-RAGE with 2μg pcDNA3-myc-S100A7 were transfected into 293T cells using Lipofectamine 2000 (Invitrogen, CA, USA) for 48 h. The cells from each dish were collected into ice-cold cell lysis buffer with protease inhibitor cocktail, incubated on ice for 30 min and store at -80°C for input control. ANTI-FLAG® M2 Affinity Gel (sigma, A2220-10ML) was added into protein lysates and incubated at 4°C overnight. Then centrifuged at 4,000rpm for 5min, at 4°C, discarded supernatant and washed gel with PBS twice, collected gel. Samples were then analyzed using western blotting according to the standard protocol described previously.

### Experimental metastasis assay *in Vivo*

Female BALB/c nude mice were bred and maintained under defined conditions at the Animal Experiment Center of Cancer Hospital, Chinese Academy of Medical sciences, and all procedures were approved by the Animal Care and Use Committee of Cancer Hospital, Chinese Academy of Medical Sciences and conformed to the legal mandates and national guidelines for the care and maintenance of laboratory animals. BALB/c female nude mice from 4-6 weeks of age were used for experimental metastasis assays, 1 × 10^6^ cells were resuspended in 100 μl PBS and injected into the lateral tail vein of mice with a 28G insulin syringe. After 15 weeks, mice were sacrificed and the lungs of the mice were harvested, fixed in 4% paraformaldehyde for further evaluation. Cryosections (4μm) of the harvested lungs were hematoxylin-eosin staining for histological assessment.

### Statistical analysis

Because of the abnormal distribution of the samples, the statistical evaluation was performed using nonparametric tests (Kruskal-Wallis Test or Mann-Whitney U test) with the SPSS software version 22.0 (SPSS Inc., Chicago, IL). Results with two-tailed *P*-values less than 0.05 were considered statistically significant.

## SUPPLEMENTARY MATERIALS FIGURES


