# Supplementary material for: S100A7 promotes the migration, invasion and metastasis of human cervical cancer cells through epithelial–mesenchymal transition
Source: Oncotarget. 2017 Feb 15;8(15):24964–77. doi: 10.18632/oncotarget.15329 (PMC5421902; doi:10.18632/oncotarget.15329)
Supplement: Supplementary file 1 [file oncotarget-08-24964-s001.pdf]

S100A7 promotes the migration, invasion and metastasis of human cervical cancer cells through epithelial–mesenchymal transition

SUPPLEMENTARY FIGURE

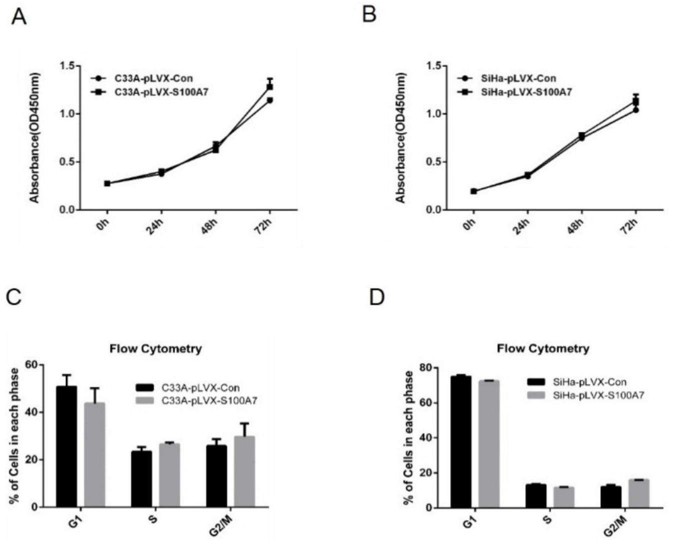

Supplementary Figure 1: S100A7 has no impact on cell proliferation and cell cycle progression of cervical cancer cells. Cell proliferation was determined by CCK-8 assay in C33A **A.** and SiHa **B.** cells. Cell-cycle distribution was analyzed by FACS in C33A **C.** and SiHa **D.** cells.
